# Work-related injuries and illnesses among kitchen workers at two major students’ hostels

**DOI:** 10.1186/s42506-024-00163-x

**Published:** 2024-07-08

**Authors:** Ghada O. Wassif, Abeer Abdelsalam, Waleed Salah Eldin, Mona A. Abdel-Hamid, Samia I. Damaty

**Affiliations:** Department of Community, Environmental & Occupational Medicine, Faculty of Medicine, Ain Shams University, Cairo, Egypt

**Keywords:** Kitchen work, Hostel, Accident, Work-related injuries, Work-related illnesses

## Abstract

**Background:**

Kitchen workers face a disproportionately high risk of injuries and illnesses. Yet, despite the ubiquity of kitchen-related injuries, there has been a relative lack of comprehensive research on this topic, particularly in developing countries. Ain Shams University, as a prestigious educational institution with its diverse kitchen staff, provides an ideal setting for studying the challenges faced in professional kitchens. This study aims to measure the prevalence of work-related injuries and illnesses among kitchen workers of two major students’ hostels at Ain Shams University in Cairo and to explore their risk factors.

**Methods:**

A cross-sectional analytical study involving kitchen staff from two hostels that house students at the university was carried out in 2021. The study involved all kitchen staff in the dorms for students (*n* = 128). Data was collected using a structured interview questionnaire.

**Results:**

The findings showed a significant prevalence of work-related illnesses (81.3%) and injuries (77.3%) among restaurant employees. Age, education, and job type all had a statistically significant relationship with the frequency of work-related injuries over the previous year. Additionally, there was a statistically significant relationship between age, job type, and the frequency of diseases among kitchen workers (*p* ≤ 0.05).

**Conclusion:**

Cooks and other food service employees are more susceptible to occupational diseases and injuries in the workplace. Restaurants and the university hostel authorities should implement preventative measures and policies to lower the risk of harm among these employees by offering occupational health and safety services such as training and education programs, personal protective equipment, and regular health examinations.

## Introduction

The Bureau of Labor Statistics indicated that the restaurant industry is notably ranked third for the frequency of workplace injuries and fifth for the number of work absences resulting from such injuries [1]. This data underscores the need for enhanced safety measures and support systems to protect employees during unprecedented times.

The International Labor Organization (ILO) estimates that millions of workplace accidents and injuries occur each year. The statistics paint a grim picture: every 15 s, a worker dies from a work-related illness or injury, and every minute, 160 workers experience a work-related accident. This results in an astounding 317 million work-related injuries each year, many of which lead to prolonged absences from work. The repercussions extend beyond the individual, with the estimated annual economic burden of substandard occupational safety and health practices amounting to 4% of global GDP [2, 3].

In the culinary sector, cooks and other kitchen staff face particularly strenuous conditions. They contend with heavy workloads and operate in challenging environments characterized by high temperatures, poor ventilation, and slippery floors, all contributing to a heightened risk of occupational diseases and accidents [4–9].

Despite the significant impact of kitchen work environments on employee health, workplace safety, and the quality of food produced, there is a notable gap in research within the Arab world. Addressing this gap is crucial, as analyzing occupational injuries and diseases is fundamental for devising effective preventative policies. This process involves identifying potential causes, tracing their origins, developing solutions, and implementing measures to safeguard the well-being of workers. This study aims to (1) measure the prevalence of work-related injuries and illnesses among kitchen workers of two major students’ hostels at a prestigious Egyptian university in Cairo and (2) explore risk factors for work-related injuries and illnesses among participating kitchen workers.

## Methods

### Study design

This cross-sectional analytical study included kitchen staff from two hostels that housed students at a prestigious Egyptian university in Cairo during the year 2021. It provides more than 4000 meals, served three times a day, 7 days a week, primarily to male and female students in the included hostels. The kitchen also serves many employees (200–300 lunches daily). Handling and storing ingredients, administrative work, food preparation, hot cooking, packing, serving, dishwashing, and cleaning were all categorized as part of the cooking process.

### Study participants

The study involved all of the kitchen staff (*n* = 128) in the dorms for students. Chief cooks (*n* = 23), assistant cooks (*n* = 22), laborers (*n* = 25), food presenters (*n* = 15), supervisors (*n* = 26), and administrative staff (*n* = 17) make up the current research group. All work full-time, 5 or 6 days a week, for at least 6 h daily.

### Study tools

Data were collected through an anonymous interview questionnaire structured into two main sections. The first section captured demographic information about the study participants, including age, gender, place of residence, educational level, marital status, and job category. The second section focused on work-related illnesses and injuries, such as cuts, burns, slips, trips, joint disorders, gastrointestinal issues, eye conditions, and respiratory, hypertensive, and dermatological diseases, with references to the relevant literature [6, 7, 10]. Only incidents from the preceding 12 months were reported, and participants responded to the questionnaire items with a simple “Yes” or “No.” The questionnaire was administered in the Arabic language.

The validation process for the translated tool involved several critical steps: forward and back translation technique to ensure linguistic accuracy during the translation from English to Arabic and to maintain semantic equivalence while adapting the tool for an Arabic-speaking audience. Experts in occupational medicine and public health meticulously reviewed the translated tool to ensure face validation; their expertise ensured that the content was contextually appropriate and aligned with the intended purpose.

Pilot testing was conducted among 20 kitchen workers; the purpose was to assess the clarity and comprehensiveness of the tool in its Arabic-translated form. Valuable insights from this pilot testing phase guided further refinements. Notably, data collected during the pilot study were excluded from subsequent analyses to avoid potential bias and ensure that the validation process remains robust.

Anthropometric measurements, including weight and height, were taken for all participating workers. The body mass index (BMI) was calculated using the formula: BMI = weight (kg)/height (m^2^). The study achieved a 100% response rate from the kitchen workers involved, as the administration of the student hostels was eager to understand the results to implement informed interventions to prevent illnesses and injuries among their staff.

### Statistical analysis

The Statistical Package for Social Sciences (SPSS) version 23.0 was used to analyze the data. The univariate relationships between the demographic characteristics (independent variables) and the reported work-related illnesses and injuries (dependent variables) were evaluated using the chi-square test. Logistic regression was used to identify the independent risk factors for injuries and illnesses among participating kitchen employees. Statistics were judged significant at *p* ≤ 0.05.

## Results

Table 1 indicated that nearly half of the participating kitchen staff were between the ages of 40 and 50 years old. A small fraction of the study participants were under the age of 30. The workforce was predominantly male, with the majority residing in Cairo, and most were married. The educational background varied, with many having completed primary, preparatory, and secondary education. When considering body mass index (BMI), more than one third were classified as overweight, while others fell into the obese grade 1 (28.9%) and grade 2 (10.2%) categories. The majority of the participants were employed in kitchen roles, with a smaller segment working in administrative capacities (13.3%). The average job duration was nearly 18 ± 9 years, with a vast majority having been in the same position for over a decade.

**Table 1:** Demographic characteristics of the participating kitchen workers at two major students’ hostels, Cairo, 2021 (*n* = 128)

Demographic characteristics	*N*	%
**Age (years)**		
20–30	4	3.1
31–40	35	27.3
41–50	63	49.2
51–65	26	20.4
Mean ± SD Range	44.8 ± 8.24 (24–59)	
**Gender**		
Male	85	66.4
Female	43	33.6
**Residence**		
Cairo	81	63.3
Outside Cairo	47	36.7
**Marital status**		
Single/widowed	9	7
Married	119	93
**Education**		
Illiterate	16	12.5
Primary/preparatory	58	45.3
Secondary	40	31.3
University/postgraduate studies	14	10.9
**Body mass index (kg/m**^**2)**^		
Normal (18.5–24.9)	30	23.4
Overweight (25–29.9)	47	36.7
Obese grade 1 (30–34.9)	37	28.9
Obese grade 2 (35–39.9)	13	10.2
Obese grade 3 (more than 40)	1	0.8
Mean ± SD Range	28.9 ± 4.7 (18.83–40.12)	
**Job**		
Kitchen-related job	111	86.7
Administrative job	17	13.3
**Job duration (years)**		
< 5 years	15	11.7
5–10 years	5	3.9
> 10 years	108	84.4
Mean ± SD Range	18 ± 9 (1–40)	

*SD* standard deviation

Table 2 indicates a statistically significant relationship between the incidence of work-related injuries in the past year and factors such as advanced age, a lower education level, and employment in kitchen-related job categories. The frequency of accidents was notably higher among those in the 41–50-year age group, individuals without formal education, and those holding positions related to kitchen work.

**Table 2:** Risk factors for work-related injuries among participating kitchen workers at two major students’ hostels, Cairo, 2021 (*n* = 128)

Variable	No accident *N* (%) Total = 29	Accident *N* (%) Total = 99	*p*-value *χ*^2^
Age (years)			
20–30	1 (25.0)	3 (75.0)	FE# 0.004*
31–40	15 (42.9)	20 (57.1)	
41–50	7 (11.1)	56 (88.9)	
51–65	6 (23.1)	20 (76.9)	
Gender			
Male	23 (27.1)	62 (72.9)	0.094
Female	6 (14.0)	37 (86.0)	
Residence			
Cairo	18 (22.2)	63 (77.8)	0.878
Outside Cairo	11 (23.4)	36 (76.6)	
Marital status			
Single/widowed	1 (11.1)	8 (88.9)	FE# 0.683
Married	28 (23.5)	91 (76.5)	
Education			
Illiterate	0 (0.0)	16 (100)	FE# < 0.001*
Primary/preparatory	3 (5.17)	55 (94.48)	
Secondary	18 (45)	22 (55.0)	
University/postgraduate studies	8 (57.1)	6 (42.9)	
Job category			
Kitchen-related jobs	18 (16.2)	93 (83.8)	< 0.001*
Administrative jobs	11 (64.7)	6 (35.3)	
Job duration (years)			
< 5	4 (26.7)	11 (73.3)	FE# 0.471
5–10	2 (40.0)	3 (60.0)	
> 10	23 (21.3)	85 (78.7)	
Body mass index (kg/m^2^)			
Normal	7 (23.3)	23 (76.7)	0.694
Overweight	11 (23.4)	36 (76.6)	
Obese grade 1	10 (27.0)	27 (73.0)	
Obese grade 2	1 (7.7)	12 (92.3)	
Obese grade 3	0 (0.0)	1 (100.0)	

#Fischer’s exact test was used as (20.0%) of the cells or more have an expected count of less than 5

*Statistically significant at *p* ≤ 0.05

Table 3 shows that gender, education, and job duration were significant independent risk factors of work-related injuries in the participating kitchen workers.

**Table 3:** Logistic regression revealing independent risk factors of work-related injuries in the participating kitchen workers

Variable	*B*	S.E	Wald	Sig	Exp(B)	95% C.I. for EXP(B)	
						**Lower**	**Upper**
**Age**	0.484	0.379	1.634	0.201	1.623	0.772	3.409
**Residence**	− 0.204	0.151	1.819	0.177	0.816	0.607	1.097
**Gender**	2.152	0.657	10.727	0.001^**^	8.600	2.373	31.168
**Education**	− 1.857	0.517	12.898	0.000^**^	0.156	.057	0.430
**Job duration**	0.941	0.441	4.562	0.033^*^	2.564	1.081	6.083
**Body mass index (BMI)**	− 0.109	0.287	0.145	0.703	0.896	0.511	1.572
**Job category**	0.322	0.910	0.125	0.723	1.380	0.232	8.207

^*^Statistically significant at *p* ≤ 0.05

^**^Highly statistically significant at *p* ≤ 0.01

Table 4 demonstrates a statistically significant relationship between the prevalence of illnesses among kitchen workers and their age and job category. The data suggest that illnesses were more frequently reported in older participants, particularly those aged 51–65 years, and among individuals employed in kitchen-related roles.

**Table 4:** Risk factors for illnesses among participating kitchen workers (*n* = 128)

Variable	Not diseased *N* (%) Total = 24	Diseased *N* (%) Total = 104	*p*-value
Age (years)			
20–30	1 (25.0)	3 (75.0)	FE# 0.035*
31–40	11 (31.4)	24 (68.6)	
41–50	11 (17.5)	52 (82.5)	
51–65	1 (3.8)	25 (96.2)	
Gender			
Male	19 (22.4)	66 (77.6)	0.142
Female	5 (11.6)	38 (88.4)	
Residence			
Cairo	13 (16.0)	68 (84.0)	FE# 0.304
Outside Cairo	11 (23.4)	36 (76.6)	
Marital status			
Single/widowed	1(11.1)	8 (88.9)	FE# 1.000
Married	23 (19.3)	96 (80.7)	
Education			
Illiterate	1 (11.1)	8 (88.9)	FE# 0.225
Primary/preparatory	8 (13.7)	50 (86.2)	
Secondary	9 (22.5)	31 (77.5)	
University/postgraduate studies	5 (35.7)	9 (64.3)	
Job category			
Kitchen-related jobs	16 (14.4)	95 (85.6)	0.001*
Administrative jobs	8 (47.1)	9 (52.9)	
Job duration (years)			
< 5 years	4 (26.7)	11 (73.3)	FE# 0.530
5–10 years	0 (0.0)	5 (100.0)	
> 10 years	20 (18.5)	88 (81.5)	
BMI			
Normal	6 (20.0)	24 (80.0)	FE# 0.389
Overweight	11 (23.4)	36 (76.6)	
Obese 1	7 (18.9)	30 (81.1)	
Obese 2	0 (0.0)	13 (100.0)	
Obese 3	0 (0.0)	1 (100.0)	

#Fischer’s exact test was used as (20.0%) of the cells or more have an expected count of less than 5

*Statistically significant at *p* ≤ 0.05

Table 5 shows that age and job category were significant independent risk factors of work-related illnesses in the participating kitchen workers.

**Table 5:** Logistic regression revealing significant independent risk factors of illnesses in the participating kitchen workers

Variable	*B*	S.E	Wald	Sig	Exp(B)	95% C.I. for EXP(B)	
						**Lower**	**Upper**
**Age**	0.983	0.409	5.773	0.016^*^	2.672	1.199	5.955
**Residence**	− 0.267	0.140	3.613	0.057	0.766	0.582	1.008
**Gender**	0.803	0.569	1.993	0.158	2.231	0.732	6.801
**Education**	0.312	0.423	0.546	0.460	1.366	0.597	3.128
**Job duration**	− 0.105	0.429	0.060	0.806	0.900	0.389	2.086
**BMI**	0.157	0.280	0.314	0.575	1.170	0.676	2.025
**Job category**	− 2.213	0.958	5.334	0.021*	0.109	0.017	0.715

^*^Significant at *p* < 0.05

## Discussion

Cooks and food service workers are at elevated risk for workplace injuries and illnesses due to exposure to various dangers in the cooking process and their intensive manual workload. More than three fourths (77.3%) of the participating kitchen workers suffered from work-related injuries. More than half (53.9%) and 57% suffered from cut wounds and skin burns, respectively. The majority (71.1%) experienced slips, trips, or falls.

The prevalence of injuries in the present study was higher than in other studies; a study done in India among cooks reported that skin burns were 40.5% and cut wounds were 33.5% [8]. Another study carried out in Japan mentioned that more than one third of food service workers reported work-related injuries [9]. In addition, the prevalence of work-related injuries among catering students in the west of Ireland was 12% [11]. In Korea, among kitchen workers, slips and falls were mainly the most frequent types of injuries (25.9%), followed by burns and scalds (22.7%) and cuts, amputations, and punctures (21.9%) [7]. Cut wounds due to using knives and broken glass objects were the most common incidents occurring to immigrant Chinese restaurant workers in the United States. Burns were the second leading cause of work-related injuries caused by working in warm environments, handling hot dishes, and exposure to hot grease and hot water [12]. The frequency of injuries among Japanese commercial kitchen workers was 23.8% for cut injuries and 15.9% for burn injuries [13].

While the results of the present study were similar to a study done among cooks and food service workers (CFSWs) in Korea [14], the order differs; the most common injuries were bruises (92.8%), followed by burns (73.0%), cuts and lacerated wounds (69.4%), sprains (47.7%), and falls (42.8%). Also, the study done in Iran revealed that the prevalence of work-related injuries among kitchen workers was 84%. Cuts (67.7%), thermal burns (63.7%), falls, slips, and being injured by a moving object (33.7%) were the leading causes of work-related injuries among restaurant workers [10]. In another study in Korea, the most common accidents were knife-cut wounds (84.7%) and burns caused by hot water and oil (74.4%), then slip and fall (28.1%) [15].

The similarities in the results of the present study with those conducted among kitchen workers in Korea [14] and Iran [10] could be attributed to several common factors inherent in the kitchen work environment. These factors include high-risk work environments: kitchens are fast-paced settings where workers are often required to handle sharp utensils, work with hot surfaces and substances, and move quickly, which increases the risk of accidents such as cuts, burns, and falls. Common occupational hazards are as follows: certain injuries like cuts from knives, burns from hot oil, and slips due to wet floors are prevalent across kitchens worldwide due to the nature of the tasks involved. Similar work practices area as follows: kitchen staff globally may share similar work practices, such as using similar tools and equipment, which can lead to comparable types of injuries.

These commonalities suggest that regardless of the geographical location, the fundamental risks associated with kitchen work remain consistent, leading to similar injury patterns observed in different studies. It underscores the importance of implementing effective safety measures and training programs to reduce these risks across the industry.

The findings of the present study revealed that work-related injuries in the last year were more common among participants with older age, lower education level, and kitchen-related job categories, mainly cooks and cooking assistants. This is consistent with the study done in Iran, where among work activities, the distribution of injured persons was significantly higher in cooking processes (51.3%), followed by preparation and washing (30%), compared to other activities [10]. The present study’s finding also agrees with the study conducted in South Korean restaurants, where cooking and food preparation were the most common causes of occupational injuries [16]. This may be along with such risk factors as exposure to hot processes (hot grease and steam), handling knives and sharp blades, walking on slippery floors, and contact with hot dishes during washing and preparation activities.

The present study showed that there was an association between older age and the occurrence of work-related injuries. This is consistent with studies done in Korea [16, 17] and France [18], where more than half of the injured kitchen workers were in their 50 s and 60 s. However, studies conducted in Korea [19], Japan [20], and Iran [10] have shown inconsistencies. These discrepancies may stem from the higher proportion of older individuals in the current study, where nearly half of the participants were between 40 and 50 years while the group under 30 years old was the smallest (3.1%). This demographic distribution could be attributed to a decrease in governmental sector employment. In contrast, the majority of subjects in similar studies among kitchen and restaurant workers were of a younger age.

The present study showed that 62.5% suffered from joint disorders; this is consistent with a study done in Iran [10] where the highest prevalence of work-related diseases was musculoskeletal disorders (70.3%). The prevalence of work-related musculoskeletal disorders (WRMSDs) in a study done in Ethiopia was very high (81.5%) [21]. Also, studies done in Japan [13, 22] and China [23] reported a high prevalence of musculoskeletal disorders.

In the current study, we investigated the prevalence of work-related skin diseases among restaurant staff. Notably, we found that 11.7% of restaurant workers experienced such conditions. Interestingly, this aligns closely with Irish research, which reported a 15% prevalence of skin disorders in Irish restaurants [11]. However, a study conducted in Iran revealed a higher rate of 24.7% for work-related skin conditions among restaurant workers in that context [10]. The elevated figures observed in the Iranian study may be attributed to frequent exposure to detergents and disinfectants—a primary contributor to dermatitis in this population. Additionally, the propensity for skin abrasions and lacerations heightens the risk of fungal infections, which thrive in the humid environment typical of restaurant kitchens. Implementing the use of protective gloves could mitigate these risks by reducing direct contact with irritant substances and safeguarding against cuts and scratches.

The prevalence of gastrointestinal (GI) diseases was less than 10%. Contrastingly, a study conducted in Iran reported a higher prevalence, with 21.3% of participants experiencing GI symptoms [10].

In this study, 17.2% of restaurant workers were found to have respiratory problems. This incidence aligns with findings from similar research conducted on restaurant workers in Iran, Thailand, and Nigeria [10, 24, 25]. The primary contributors to these respiratory conditions are exposure to cooking fumes and a spectrum of organic compounds, including formaldehyde, acrolein, and total volatile organic compounds (TVOCs). Additionally, the situation is aggravated by the presence of suspended particulate matter in the ambient air which further exacerbates the problem [26].

The current study indicates that fewer than 10% of kitchen workers experienced eye disorders, such as irritation, blurred vision, and eye fatigue. This prevalence is notably lower compared to a study in Iran, which found that 29% of restaurant workers reported eye-related symptoms [10]. In contrast, comprehensive eye examinations among kitchen staff in Ghana revealed a much higher incidence, with 75.2% suffering from various eye conditions [27]. The lower rate observed in the present study may be attributed to the fact that not all participants were exposed to cooking steam, a known risk factor for eye disorders.

### Limitations

This study has certain limitations that warrant careful consideration when interpreting its findings. The cross-sectional design and the subjective nature of self-reported data may preclude definitive causal inferences. Additionally, the reliance on a 12-month retrospective questionnaire could introduce recall bias, potentially influencing the accuracy of the results. Despite these constraints, the data measured and the correlations observed with work environment, health, and demographic factors are consistent with existing literature. It is also important to note that detailed information regarding the severity of injuries or the extent of damage from accidents was not available for analysis.

## Conclusion

The results of this study highlight a significant occurrence of work-related injuries and illnesses among kitchen workers, with reported rates of 77.3% and 81.3%, respectively. Given these alarming statistics, it is crucial to adopt comprehensive preventive strategies and policies. These should include the provision of occupational health and safety services, encompassing safety practice training, the use of personal protective equipment, and regular medical examinations to monitor workers’ health. Additionally, there is an urgent need for in-depth research to accurately determine the incidence, characteristics, and causes of work-related injuries and diseases within Egypt’s catering sector.

To deepen our understanding of work-related injuries and illnesses among kitchen workers, detailed environmental studies of kitchen workplaces are recommended. These studies should assess factors such as floor conditions, materials, ventilation systems, and ergonomic design. The insights gained from such research would be invaluable in aiding authorities to formulate and implement an effective health and safety management system, thereby improving the safety and welfare of kitchen personnel.

## Data Availability

Data are available from the corresponding author upon request.

## Abbreviations

BMI: Body mass index
CFSWs: Cooks and food service workers
FSWs: Food service workers
GDP: Gross domestic product
Hr/wk.: Hour per week
ILO: International Labor Office
SPSS: Statistical Package for Social Sciences
*p*: *p*-Value
SD: Standard deviation
χ^2^: Chi-square
CI: Confidence interval
S.E.: Standard error
WRMSDs: Work-related musculoskeletal disorders
PNS: Peripheral nervous system
CNS: Central nervous system
TVOCs: Total volatile organic compounds
COVID-19: Coronavirus disease 2019
